# Prevalence and Estimation of the Evolution of Myopia in Spanish Children

**DOI:** 10.3390/jcm13061800

**Published:** 2024-03-21

**Authors:** Miguel Ángel Sánchez-Tena, Clara Martinez-Perez, Cesar Villa-Collar, Mariano González-Pérez, Ana González-Abad, Grupo de Investigación Alain Afflelou, Cristina Alvarez-Peregrina

**Affiliations:** 1 Optometry and Vision Department, Faculty of Optics and Optometry, Complutense University of Madrid, 28040 Madrid, Spain; masancheztena@ucm.es (M.Á.S.-T.); marian06@ucm.es (M.G.-P.); 2 School of Management, Engineering and Aeronautics, ISEC LISBOA (Instituto Superior de Educação e Ciências), 1750-179 Lisbon, Portugal; clara.perez@iseclisboa.pt; 3 Faculty of Biomedical and Health Science, Europea University of Madrid, 28670 Madrid, Spain; villacollarc@gmail.com; 4 Training and Development Department, Alain Afflelou Óptico, 28046 Madrid, Spain; a.glezabad@hotmail.com (A.G.-A.); adiaz@afflelou.es

**Keywords:** myopia, childhood, Spain, prevalence, estimations

## Abstract

**Background**: In recent decades, myopia has turned into a public health issue across the world. Between 1993 and 2016, the percentage of myopes increased from 10.4% to 34.2%. This study aims to analyze the myopia prevalence in Spanish children from five to seven years old over the last five years and to predict the rate of myopia in Spain by 2030. **Methods**: The sample consisted of children aged between 5 and 7, who underwent an optometric examination consisting of the measurement of visual acuity (VA) and determination of objective and subjective refraction. The cut-off points to define refractive error are established by the spherical equivalent (SE) value: hyperopia for an SE over or equal to +0.50 D; myopia for an SE under or equal to −0.50 D; and emmetropia when the SE is between −0.50 D and +0.50 D. **Results**: The myopia rate in Spanish children aged between five and seven was 19%. Myopia prevalence progressively increased as children grew up (*p* ≤ 0.001). It is estimated that, in the year 2030, the rate of myopia will be 30.2% [CI: 25.3–35.0], of which 81.9% [CI: 78.3–85.3] will have low myopia, 10.0% [CI: 7.2–12.8] moderate myopia, and 7.3% [CI: 4.9–9.7] high myopia. **Conclusions**: Nineteen percent of Spanish children between five and seven are myopes. In 2030, is expected that 30.2% of Spanish children between 5 and 7 years of age will be myopes. These estimations will support eye care professionals in recommending that children spend more time outdoors to prevent the onset of myopia and the use of methods to control myopia to avoid reaching these figures of high myopia.

## 1. Introduction

As a result of the impact of environmental factors, myopia is reaching epidemic proportions at great speed. Myopia causes some ocular problems and increases the probability of suffering from other ophthalmologic pathologies, such as myopic retinopathy, retinal detachment, glaucoma, or cataracts [1]. Nowadays, myopia is already affecting one-third of the worldwide population, although its frequency depends on the geographical region. Myopia prevalence is higher in developed countries, particularly in East Asia, and most evidence suggests that this prevalence is increasing. In the most developed areas of East Asia, myopia affects 80–90% of young adults, while high myopia prevalence within this area is 20% [2]. This, therefore, shows that in East Asian countries, myopia rates are much higher than in other countries (East Asia: 65.5 to 96.5%; other countries: 2.8 to 35.0%). Likewise, the myopia rates among children of school age are significantly higher in the urban countries of East Asia (50 to 62% among children aged 12 years) than it is within this same age group in other countries (6 to 20% among children aged 12 years) [3]. In turn, it has been suggested that the percentage of visually impaired children will increase by 26% by the year 2060, with uncorrected refraction errors being the cause in 69% of the cases [4]. Intending to predict which children will end up developing myopia, the International Myopia Institute established the concept of pre-myopia, defined as: “a refractive state of an eye of 0.75 D and >0.50 D in children where a combination of baseline refraction, age, and other quantifiable risk factors provide a sufficient likelihood of the future development of myopia to merit preventative interventions” [5].

The prevalence of myopia in children has been linked to prolonged periods of reading, studying, and the use of digital devices, influenced by the rapid development of the modern economy and digitalization. Sherwin et al. [6] found that children working closer than 30 cm from their task had a significantly higher risk of developing myopia compared to those working at a considerably longer distance. Similarly, extended reading sessions and close-distance work have been associated with an increased risk of myopia in studies from Australia [7]. The correlation between myopia and higher education levels suggests that the demands of educational activities, requiring closer work, might contribute to the development of myopia, although the exact cause remains unclear [8]. Studies on the relationship between near vision activities and myopia have yielded mixed results, with some finding a significant correlation and others not [9,10]. The role of environmental factors, especially the lack of exposure to sunlight, has also been highlighted in the context of myopia development. Research, including the SAVES Study [11] and the Avon Longitudinal Study of Parents and Children [12], has demonstrated the protective effect of outdoor time against myopia, showing that myopic children spend less time outdoors compared to their non-myopic peers. Differences in sunlight exposure between children in different regions, such as Australia and Singapore, have been noted, further emphasizing the environmental influence on myopia [13].

In 2016, Holden et al. [1] performed a worldwide systematic review and a meta-analysis of the rates of myopia and high myopia to make an estimation of the trends over time between 2000 and 2050. They included 145 studies with 2.1 million participants, and the results estimated that in the year 2000, there were 1.406 billion myopes globally, of which 163 million had high myopia. They calculated that by 2050, there will be more than 4.758 billion, that is, half the global population, of which 938 million will have high myopia. However, myopia prevalence differs in different countries, so estimations will vary geographically. Children with myopia at a young age have the highest risk, given that the duration of the condition is longer, of developing high myopia and thus also myopic macular degeneration. For this reason, the starting age and its progression are the main indicators of the risk that the child could develop high myopia [14].

The first study to analyze the pediatric prevalence of myopia in Spain was the one conducted by Montés-Micó et al. [15] in 2000. This study found the myopia prevalence among children of 3–8 years to be 2.5%, increasing to 25.7% for the 9–19-year-old age group. This research group has analyzed myopia prevalence in children of school age (five to seven years old) since 2016. Thus, it has been found that the percentage of myopia has gone from 19.1% to 20% between 2016 and 2020 [16,17]. In recent years, children have gone through several lockdown periods due to the SARS-CoV-2 pandemic, which has required them to spend longer time at home and shorter time outdoors. Recent studies have found a significant association between myopic progression and policies during the pandemic by governments [18]. Therefore, this study aims to evaluate the rate of myopia in Spanish children aged from five to seven over the last five years, and to predict the evolution of myopia in Spain by 2030.

## 2. Materials and Methods

### 2.1. Definition of Variables

The spherical equivalent, defined as SE = sphere + (cylinder/2), was used to establish the refractive error. The classification was as follows: emmetropia: SE between −0.50 D and +0.50 D; hyperopia: SE over or equal to +0.50 D; and myopia: SE less than or equal to −0.50 D. Myopia was subclassified as follows: low: between −0.50 D and −3.00 D; medium: between −3.25 D and −6.00 D; or high: less than or equal to −6.00 D, following the classification of the American Academy of Optometry [19] and International Myopia Institute [20].

### 2.2. Clinical Procedure

First, a cross-sectional study was carried out on the prevalence of myopia in Spain between the years 2016 and 2021. Children from 5 to 7 years of age were recruited by convenience sampling from opticians from different autonomous communities in Spain.

The Research Ethics Committee of the European University of Madrid (CEI-UE) approved this research under the code CIPI/19/102. The research adheres to the principles of the Declaration of Helsinki. Parental consent was required, and they signed the informed consent form.

Inclusion criteria were ages between 5 and 7 years old.

In terms of the exclusion criteria, participants that failed to collaborate properly and cases in which the forms and survey were not filled in correctly or were incomplete were excluded.

The visual exam was performed, consisting of visual acuity, objective refraction through Mohindra retinoscopy, subjective refraction, and evaluation of the anterior segment with the slit lamp.

### 2.3. Data Analysis

The software used for carrying out the statistical analysis was SPSS 27.0 software (SPSS Inc., Chicago, IL, USA). To determine the normality of the variables, the Kolmogorov–Smirnov test was used. The Kruskal–Wallis test was used to analyze the statistical significance of the variables, considering a cut-off point of *p* ≥ 0.05.

To verify that the variables were independently associated with variable myopia and the spherical equivalent, binary and linear logistic regression analyses were performed, respectively. The criteria used to select the variables that were included in the model were those that were statistically significant in a previously performed univariate analysis or those that were clinically relevant.

Secondly, an analysis of the estimated prevalence of myopia between the years 2016 and 2030 has been carried out. Estimations were calculated through a predictive model, considering the Spanish population and the myopia prevalence in children aged 5 to 7. To carry out the predictions in the adjustment measures, the Ljung–Box statistic, the number of outliers, stationary R square, root mean square of the errors, maximum absolute percentage error, and goodness of fit have been calculated. In turn, the confidence interval of the predictions and the fitted values have been obtained.

For the forecast function in SPSS, the time series modeler approximates exponential smoothing. In addition, it estimates univariate autoregressive integrated moving average (ARIMA) and multivariate ARIMA models for time series and produces forecasts for vehicle population data.

The SPSS Forecast Modeler automatically identifies and estimates the ARIMA or exponential smoothing model that best fits the vehicle population data (series of dependent variables).

Assumptions for the forecast in SPSS are as follows:

- The data for vehicle population (dependent variable) and years (independent variable) are time series, which means that each case represents a point in time separated by a constant time interval.
- The data used are assumed to be stationary.
- The independent variables do not have missing values in the period used for the estimation.

To ensure the accuracy and robustness of our estimation model, we utilized a comprehensive statistical approach, including predictive modeling and the Ljung–Box statistic for checking randomness, alongside measures such as stationary R-square and root mean square errors to evaluate model fit.

## 3. Results

### 3.1. Demographic Data

A total of 15,672 Spanish children participated in this study, but 1994 were excluded for not fulfilling the inclusion criteria. Therefore, the studied sample comprised 14,629 participants. In Table 1, demographic data are shown based on age and gender.

### 3.2. Prevalence Results

**Figure 1:**
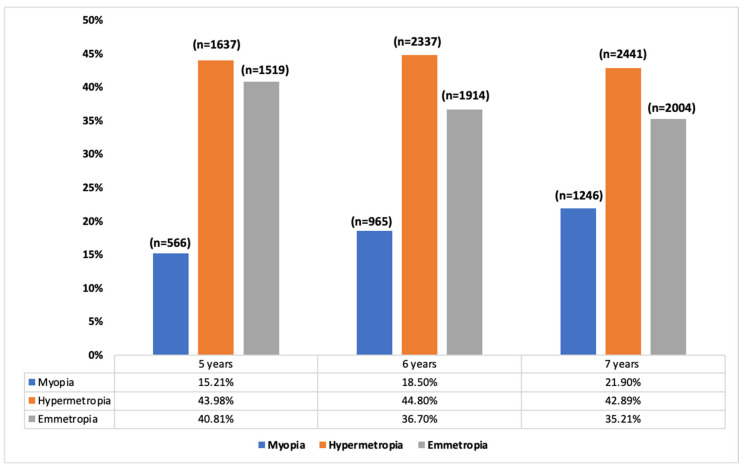
Percentage of myopes, hyperopes, and emmetropes by age.

The myopia rates in children between five and seven years old were 19%, hyperopia 44.2%, and emmetropia 36.8%. Figure 1 shows the refractive error prevalence according to age. With regards to the SE, there was a decrease in these values as age increased (*p* < 0.001). So, at five years of age, the mean value of SE was 0.84 ± 2.10 D (median [IQR]: 0.00 [1.75]), at six years it was 0.74 ± 1.99 D (median [IQR]: 0.12 [1.50]), and at seven years it was 0.60 ± 1.97 D (median [IQR]: 0.99 [1.75]). There were no differences between gender and refractive error (*p* > 0.05).

**Figure 2:**
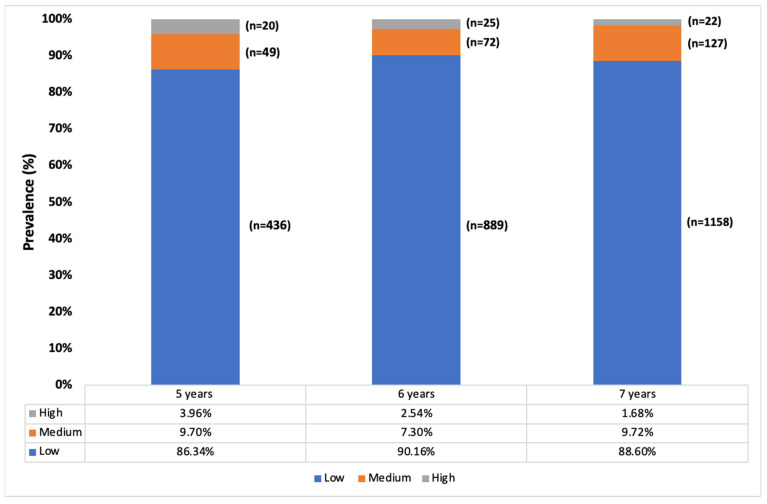
Classification of the degree of myopia by age (low: between −0.5 and −3 D; medium: between −3 and −6 D; and high: >−6 D).

In the specific demographic of myopes within the studied population, we observed that 88.7% (n = 2483) had low myopia, 8.8% (n = 248) had medium myopia, and 2.4% (n = 67) had high myopia. It is important to note that significant age-related differences in the degree of myopia were found only in the transition from five to six years old (*p* < 0.001). During this period, the prevalence of low myopia increased by 3.8% (95% CI: 1.2–6.5), while medium myopia decreased by 2.4% (95% CI: 0.01–4.7), and high myopia remained unchanged (95% CI: 0.00–2.9; *p* > 0.05). For children between six and seven years old, these variations were not statistically significant (*p* < 0.05). This suggests that the age range of five to six years is a critical period where changes in myopia degrees are more pronounced. The underlying reasons for these age-specific differences could be due to the rapid ocular growth that occurs at this young age, combined with environmental and possibly educational influences that begin to affect children as they enter more structured learning environments. Figure 2 shows the prevalence of the different degrees of myopia by age.

### 3.3. Estimations of Myopia Prevalence

**Figure 3:**
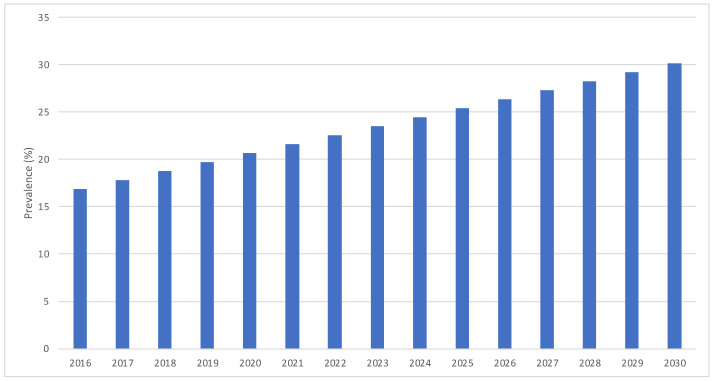
Predictive model estimations of myopia prevalence in Spain from 2016 to 2030.

**Figure 4:**
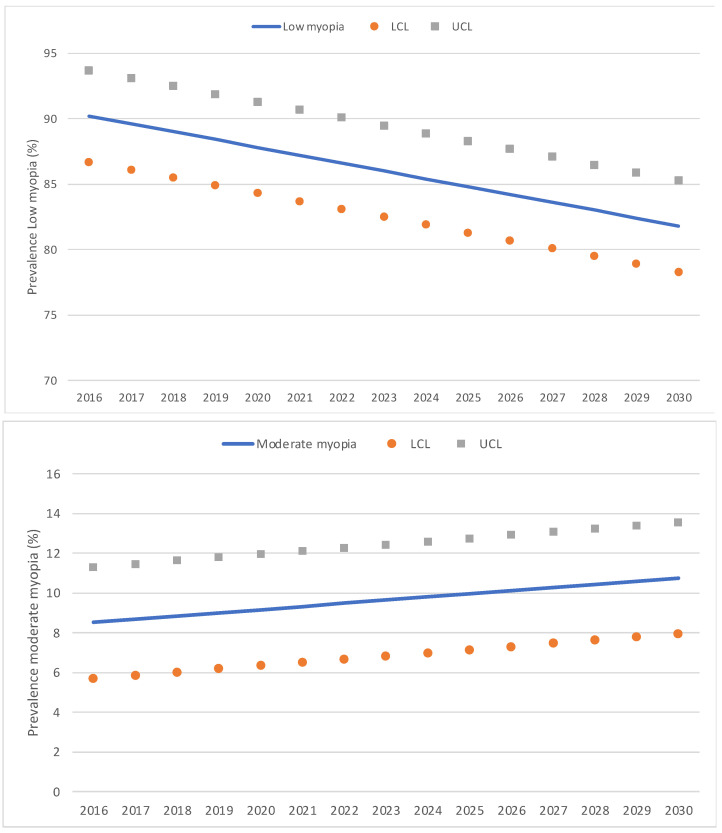

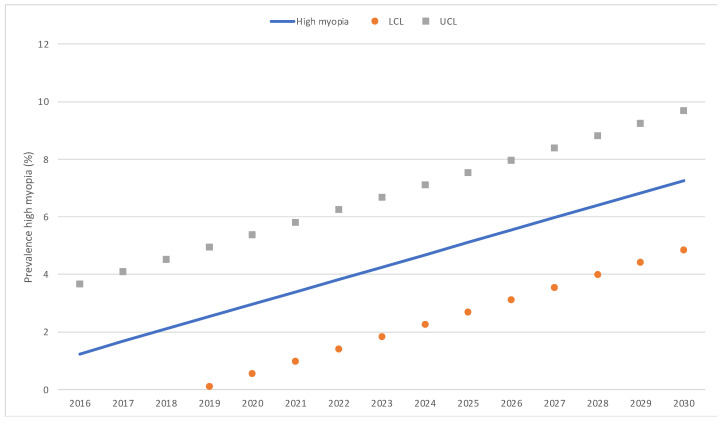
Predictive model estimations of the annual percentage of low, moderate, and high myopia. LCL: lower control limits; UCL: upper control limits.

In the trend analysis, estimations based on a predictive model indicate that by 2025, the rate of myopia will grow to 25.4% [CI: 20.6–30.3], and by 2030, it will grow to 30.2% [CI: 25.3–35.0] (Figure 3). Thus, between 5 and 7 years of age, in the year 2025, the number of children with myopia in Spain would be 264,173 [CI: 203,119–325,226], and in the year 2030, it would be 264,173 [CI: 203,053–325,293]. As for the rate of low myopia, we can predict that it would decrease, so it would be 84.8% of the myopes by 2025 [IC: 81.3–88.3] and 81.8% by 2030 [IC: 78.3–85.3]. The rate of moderate myopia also would be constant, so by 2025, it would be 10.0% of the myopes [IC: 7.2–12.8], and by 2030, it would be 10.8% [IC: 8.0–13.6]. On the other hand, it is foreseen that the rate of high myopia would increase, so by 2025, it would be 5.1% of all the myopes [IC: 2.7–7.5], and by 2030, it would be 7.3% [IC: 4.8–9.7]. Figure 4 illustrates predictive model estimations by degree of myopia.

## 4. Discussion

This study is the first to collect data of Spanish children for five years, analyzing the myopia prevalence and some of the risk factors related to myopia. The study shows that, like in other countries, there has been an increase in the myopia rates in Spain. Thus, from 2016 to 2021, the percentage of myopic children increased from 16.8% to 19.7%. It estimates that, if the number of hours in near vision continue increasing and children spend less time outdoors, by 2030, 30.2% of children aged between 5 and 7 may be myopes.

Our research findings present a lower prevalence of myopia compared to global trends, echoing the results from Holden et al.’s study [1]. This disparity primarily reflects the widespread prevalence of myopia in Asia [21,22,23,24] and the younger age demographic of our study group. Despite limited recent studies in Europe on this condition, research from London indicates an increase in myopia prescription rates from 24% to 32% between 2008 and 2017 [24]. Contrary to Holden et al.’s [1] predictions of a 36.7% myopia prevalence in our age group by 2020, our data reveal a significantly lower rate, without notable changes over the past year. This stability is unexpected, especially considering the extended indoor periods due to COVID-19 lockdowns, which led to increased near-vision activities and reduced outdoor time. Previous research in Spain has shown a decrease in the spherical equivalent (SE) during this period, suggesting a rise in myopia and pre-myopia levels among children aged five to seven [25]. Similarly, studies, including one by Wang et al. [26], have documented a 1.2- to 3-fold increase in myopia prevalence post-pandemic, particularly among children aged 6 to 8. In Hong Kong, myopia incidence surged from 11.63% pre-COVID-19 to 29.68% post-pandemic, and this rise is attributed to decreased outdoor activities during the lockdowns [27].

Comparing our findings with global data, the 19% myopia prevalence among Spanish children aged five to seven, from 2016 to 2021, stands in stark contrast to higher rates observed in Asian countries and among populations considered at high risk for myopia [21,22,23,24]. This discrepancy likely arises from a combination of cultural practices, visual habits, and genetic predispositions. Our results, when viewed alongside studies like those by Chen M. et al. [22] in Fenghua City, China, and Wong K et al. [24] in London, emphasize the significant impact of environmental and lifestyle factors on myopia prevalence across different regions. The studies in Asia, for example, not only highlight the rapid increase in myopia among youth due to intensive educational demands and limited outdoor activity but also underline the need for targeted public health strategies. The research from London further supports the theory that urban lifestyles, characterized by prolonged indoor activities and screen time, contribute to the rising trend in myopia. These findings collectively underscore the complex interplay between genetics, environment, and lifestyle in shaping myopia trends globally, urging a nuanced approach to understanding and addressing this growing public health concern.

Concerning the growth of myopia that has been forecast in our results, it matches Holden et al.’s study [1]. This is one of the biggest challenges for eye care professionals since they need to know the children that may become myopes or whose myopia is growing rapidly. Chua et al. [14] proved that age is the best predictor of developing myopia and of high myopia. Nevertheless, Williams et al. [28] found that age only accounts for 15% of growth in myopia.

As previously demonstrated, the progression of high myopia is higher than that of moderate myopia. Thus, in the study by Kumar Verkicharla et al. [29], they have already shown that in moderate myopes under 15 years of age, the rate of myopia progresses by 8%. The reason for this decrease in the rate of moderate myopia may be that there are more and more methods to control the evolution of myopia, and therefore people have greater access to these methods. Comparing our results on the estimation of myopia prevalence by 2030 with the studies by Holden et al. [1] and others reveals some key similarities and differences in terms of trends and implications for the future. According to the study by Holden et al. published in Ophthalmology in 2016, the global prevalence of myopia is projected to increase significantly, from 1406 million people (22.9% of the global population) in 2000 to 4758 million (49.8% of the global population) by 2050. This study also predicts a considerable increase in the prevalence of high myopia, suggesting a scenario where almost 10% of the global population could be affected by this condition by 2050, representing a significant risk of permanent visual disability.

Comparing this with our results, which predict an increase in the prevalence of myopia among Spanish children aged five to seven years to 30.2% by 2030, it is observed that although our estimates focus on a specific demographic and geographic group and a closer temporal horizon, both datasets point to a concerning growing trend in the prevalence of myopia. A notable difference is the scope and scale of the projections. While our results focus on a specific increase within a country and in a specific age group by 2030, the cited studies encompass global projections until 2050, suggesting a dramatic increase in the prevalence of myopia and high myopia worldwide. This underscores the importance of localized intervention strategies, tailored to the specific needs of populations and regions, while considering global trends for a comprehensive understanding of myopia as an emerging public health challenge. Looking towards the future, it is expected that the prevalence of myopia will continue to increase, but at a rate that could be moderated by improved and broader access to effective treatments. Ongoing research and the development of new treatment strategies, such as specialized contact lenses, pharmacological therapies, and refined surgical procedures, represent a beacon of hope for controlling the escalation of myopia.

The comparison of the results obtained from our study with research on the post-COVID-19 impact reveals diversity in responses to the pandemic and its effects on visual health worldwide. While in Hong Kong [30], a significant increase in the prevalence of myopia among children was observed following COVID-19 restrictions, jumping from 23.5%–24.9% pre-pandemic to 36.2% in 2021, in Xuzhou, China [31], the prevalence remained relatively stable, with a slight increase from 3.1% in 2019 to 3.5% in 2021. This contrast points to fundamental differences in confinement measures, access to outdoor spaces, and cultural and educational practices between regions. Our study shows a growing trend in the prevalence of myopia, projecting an increase from 19% to 30.2% by the year 2030. This increase, although significant, does not reach the levels observed in Hong Kong, suggesting that local conditions, including pandemic management and lifestyle changes, can have a profound impact on the development of myopia. The relative stability observed in Xuzhou further suggests that certain mitigation practices or environmental factors may have played a role in counteracting the potential increase in myopia prevalence due to the pandemic. The significant variability in the incidence and progression of myopia among these studies underscores the importance of considering the specific factors of each context when developing prevention and control strategies.

The recent surge in myopia cases among children has underscored the necessity for early intervention strategies and robust public health policies aimed at mitigating this trend. Firstly, the significant rise in myopia prevalence, especially in East and Southeast Asia, but also increasingly recognized globally, calls for a multifaceted approach to control. Strategies like increasing outdoor activity time for children have been consistently advocated for, based on evidence suggesting its effectiveness in preventing the onset of myopia. Such interventions are not only clinically beneficial but also enhance overall physical health and well-being, thereby representing a holistic public health strategy [29].

Furthermore, the application of clinical interventions to slow myopia progression, such as the use of low-dose atropine eye drops, orthokeratology lenses, ophthalmic lenses, and soft contact lenses for myopia management, has been supported by a wealth of research. These treatments offer promising avenues for controlling myopia progression when applied early and tailored to individual needs based on factors like age, ethnicity, and myopia progression rate. Each of these interventions comes with its considerations, such as potential side effects, costs, and treatment complexity, necessitating informed decision making by healthcare providers and families [32,33]. Additionally, the exploration of the combined effects of various interventions marks an emerging area of interest. This integrated approach, considering both lifestyle modifications and clinical treatments, could potentially amplify the effectiveness of myopia control strategies. It emphasizes the need for personalized care plans that account for the unique risk factors and circumstances of each child, aiming to optimize outcomes and minimize the risk of myopia progression and its complications [32,33].

As previously mentioned, the greatest strength of this study was the inclusion of a large number of subjects and the resulting data which made it possible to make annual estimations for myopia prevalence. So far, this is the biggest sample of data that has been used to determine myopia prevalence in a Spanish population. However, this study has certain limitations. To select the sample, randomization was not used, and it was carried out through convenience sampling. In addition, glasses were given to those children who needed them. On the other hand, we must make use of non-cycloplegic refraction, due to the fact that nowadays in Spain, the use of diagnostic drugs by optometrists is forbidden. However, these limitations are compensated for, given the extent of the sample size to estimate the myopia prevalence. In addition, regarding the use of cycloplegia, we used as a reference the studies published in IOVS in its special White Papers by the International Myopia Institute (IMI) [34]. In this special issue, myopia has been defined and instructions about how to carry out clinical studies have been given. Thus, in the article by Gifford et al., “IMI—Clinical Management Guidelines Report”, the Standard Procedure for Examination is defined, with step 2 being as follows: Refraction: non-cycloplegic and/or cycloplegic refraction as indicated. Our approach aligns with established research, indicating that non-cycloplegic measurements are a reliable alternative for epidemiological studies focused on refractive errors in children. However, according to what is reported by the International Myopia Institute [20], care should be taken that both eyes are used in a study; the use of “either eye” to define myopia means that some hyperopic or emmetropic eyes can be included in a study population of myopes. For this reason and to avoid classifying hyperopic patients within the myopic group, we have used only the right eye. Regarding the estimation of the prevalence of myopia, we would like to highlight that it is based only on the data obtained from the past five years. However, during these years, children went through a time of confinement due to COVID-19. That year, the spherical equivalent became more negative, which led us to think that the prevalence of myopia would grow significantly [17]. Despite this, as proven in 2021, the growth continued in the same proportion as in times with no confinement. This enables us to predict the prevalence of myopia until 2030, assuming that similar situations to COVID-19 may arise.

## 5. Conclusions

The prevalence of myopia in Spanish children aged five to seven was 19% from 2016 to 2021, with projections indicating an increase to 30.2% by 2030. This trend underscores the critical need for comprehensive prevention and control programs tailored to Spanish children’s specific needs. The importance of outdoor activities is highlighted for both preventing myopia and enhancing overall well-being, suggesting a shift towards encouraging active breaks in educational settings and awareness campaigns for parents and caregivers about the benefits of outdoor time. With the rise in digital device usage from a young age, establishing guidelines for responsible use is essential to mitigate eye strain and myopia risk. Regular eye examinations for early detection and intervention, alongside educational initiatives on myopia risks and prevention, are vital for informed health decisions. Future research should delve into the genetic, environmental, and lifestyle factors contributing to myopia, evaluating the efficacy of new preventive and corrective measures. This scenario calls for a collaborative effort among health professionals, educators, parents, and policymakers to develop and implement effective strategies, highlighting the urgency of addressing the increasing myopia rates among future generations through tailored programs and sustained research commitment.

## Figures and Tables

**Table 1:** Demographic data based on age and gender.

	Total	Male	Female
n (%)	14,629	7647 (52.3%)	6982 (47.7%)
Age			
Mean ± SD	6.14 ± 0.79	6.15 ± 0.80	6.13 ± 0.79
Median [IQR]	6 [1]	6 [2]	6 [2]

## Data Availability

The data presented in this study are available on request from the corresponding author. The data are not publicly available due to children’s data protection.

## References

[1] Holden B.A., Fricke T.R., Wilson D.A., Jong M., Naidoo K.S., Sankaridurg P., Wong T.Y., Naduvilath T.J., Resnikoff S. (2016). Global Prevalence of Myopia and High Myopia and Temporal Trends from 2000 through 2050. Ophthalmology.

[2] Xiong S., Sankaridurg P., Naduvilath T., Zang J., Zou H., Zhu J., Lv M., He X., Xu X. (2017). Time spent in outdoor activities in relation to myopia prevention and control: A meta-analysis and systematic review. Acta Ophthalmol..

[3] Ang M., Wong T.Y. (2020). Updates on Myopia: A Clinical Perspective.

[4] Varma R., Tarczy-Hornoch K., Jiang X. (2017). Visual Impairment in Preschool Children in the United States: Demographic and Geographic Variations From 2015 to 2060. JAMA Ophthalmol..

[5] Flitcroft D.I., He M., Jonas J.B., Jong M., Naidoo K., Ohno-Matsui K., Rahi J., Resnikoff S., Vitale S., Yannuzzi L. (2019). IMI—Defining and Classifying Myopia: A Proposed Set of Standards for Clinical and Epidemiologic Studies. Investig. Ophthalmol. Vis. Sci..

[6] Sherwin J.C., Reacher M.H., Keogh R.H., Khawaja A.P., Mackey D.A., Foster P.J. (2012). The association between time spent outdoors and myopia in children and adolescents: A systematic review and meta-analysis. Ophthalmology.

[7] Ip J.M., Saw S.M., Rose K.A., Morgan I.G., Kifley A., Wang J.J., Mitchell P. (2008). Role of near work in myopia: Findings in a sample of Australian school children. Investig. Ophthalmol. Vis. Sci..

[8] Williams K.M., Bertelsen G., Cumberland P., Wolfram C., Verhoeven V.J., Anastasopoulos E., Buitendijk G.H., Cougnard-Grégoire A., Creuzot-Garcher C., Erke M.G. (2015). Increasing Prevalence of Myopia in Europe and the Impact of Education. Ophthalmology.

[9] Huang H.M., Chang D.S., Wu P.C. (2015). The Association between Near Work Activities and Myopia in Children-A Systematic Review and Meta-Analysis. PLoS ONE.

[10] Jones L.A., Sinnott L.T., Mutti D.O., Mitchell G.L., Moeschberger M.L., Zadnik K. (2007). Parental history of myopia, sports and outdoor activities, and future myopia. Investig. Ophthalmol. Vis. Sci..

[11] French A.N., Morgan I.G., Mitchell P., Rose K.A. (2013). Risk factors for incident myopia in Australian schoolchildren: The Sydney adolescent vascular and eye study. Ophthalmology.

[12] Shah R.L., Huang Y., Guggenheim J.A., Williams C. (2017). Time Outdoors at Specific Ages During Early Childhood and the Risk of Incident Myopia. Investig. Ophthalmol. Vis. Sci..

[13] Read S.A., Vincent S.J., Tan C.S., Ngo C., Collins M.J., Saw S.M. (2018). Patterns of Daily Outdoor Light Exposure in Australian and Singaporean Children. Transl. Vis. Sci. Technol..

[14] Chua S.Y.L., Sabanayagam C., Cheung Y.B., Chia A., Valenzuela R.K., Tan D., Wong T.Y., Cheng C.Y., Saw S.M. (2016). Age of Onset of Myopia Predicts Risk of High Myopia in Later Childhood in Myopic Singapore Children. Ophthalmic Physiol. Opt..

[15] Montes-Micó R., Ferrer-Blasco T. (2000). Distribution of refractive errors in Spain. Doc. Ophthalmol..

[16] Alvarez-Peregrina C.C., Sanchez-Tena M.A., Martinez-Perez C.C., Villa-Collar C.C. (2019). Prevalence and Risk Factors of Myopia in Spain. J. Ophthalmol..

[17] Alvarez-Peregrina C., Martinez-Perez C., Villa-Collar C., González-Pérez M., González-Abad A., Sánchez-Tena M.Á. (2021). The Prevalence of Myopia in Children in Spain: An Updated Study in 2020. Int. J. Environ. Res. Public Health.

[18] Watcharapalakorn A., Poyomtip T., Tawonkasiwattanakun P. (2022). Coronavirus disease 2019 outbreak and associated public health measures increase the progression of myopia among children and adolescents: Evidence synthesis. Ophthalmic Physiol. Opt..

[19] American Optometric Association (2018). Care of the Patient with Myopia.

[20] Gifford, Myopia Profile: An Information Resource for Optometrists. https://www.myopiaprofile.com/product/myopia-risk-assessment/.

[21] Li Y., Liu J., Qi P. (2017). The Increasing Prevalence of Myopia in Junior High School Students in the Haidian District of Beijing, China: A 10-Year Population-Based Survey. BMC Ophthalmol..

[22] Chen M., Wu A., Zhang L., Wang W., Chen X., Yu X., Wang K. (2018). The Increasing Prevalence of Myopia and High Myopia among High School Students in Fenghua City, Eastern China: A 15-Year Population-Based Survey. BMC Ophthalmol..

[23] Lin L.L., Shih Y.F., Hsiao C.K., Chen C.J. (2004). Prevalence of myopia in Taiwanese schoolchildren: 1983 to 2000. Ann. Acad. Med. Singap..

[24] Wong K., Dahlmann-Noor A. (2020). Myopia and its progression in children in London, UK: A retrospective evaluation. J. Optom..

[25] Alvarez-Peregrina C., Martinez-Perez C., Villa-Collar C., Andreu-Vázquez C., Ruiz-Pomeda A., Sánchez-Tena M.Á. (2021). Impact of COVID-19 Home Confinement in Children’s Refractive Errors. Int. J. Environ. Res. Public Health.

[26] Wang J., Li Y., Musch D.C., Wei N., Qi X., Ding G., Li X., Li J., Song L., Zhang Y. (2021). Progression of Myopia in School-Aged Children After COVID-19 Home Confinement. JAMA Ophthalmol..

[27] Zhang X., Cheung S.S.L., Chan H.N., Zhang Y., Wang Y.M., Yip B.H., Kam K.W., Yu M., Cheng C.Y., Young A.L. (2022). Myopia incidence and lifestyle changes among school children during the COVID-19 pandemic: A population-based prospective study. Br. J. Ophthalmol..

[28] Williams K.M., Hysi P.G., Nag A., Yonova-Doing E., Venturini C., Hammond C.J. (2013). Age of Myopia Onset in a British Population-Based Twin Cohort. Ophthalmic Physiol. Opt..

[29] Verkicharla P.K., Kammari P., Das A.V. (2020). Myopia progression varies with age and severity of myopia. PLoS ONE.

[30] Zhang X.J., Zhang Y., Kam K.W., Tang F., Li Y., Ng M.P.H., Young A.L., Ip P., Tham C.C., Chen L.J. (2023). Prevalence of Myopia in Children Before, During, and After COVID-19 Restrictions in Hong Kong. JAMA Netw Open..

[31] Li Q., Zhou W., Liao Y., Chen H., Sun Y., Wang M., Wang X., Wang W. (2023). Prevalence Trend of Myopia during the Post-COVID-19 Epidemic Period among Preschoolers: A Prospective School-based Study. Optom. Vis. Sci..

[32] Nucci P., Liu S.H., Villani E. (2023). Cochrane corner: Interventions for myopia control in children. Eye.

[33] Zhang G., Jiang J., Qu C. (2023). Myopia prevention and control in children: A systematic review and network meta-analysis. Eye.

[34] Gifford K.L., Richdale K., Kang P., Aller T.A., Lam C.S., Liu Y.M., Michaud L., Mulder J., Orr J.B., Rose K.A. (2019). IMI—Clinical Management Guidelines Report. Investig. Ophthalmol. Vis. Sci..

